# The Influence of Intolerance of Uncertainty on Social Anxiety in University Students: The Sequential Mediating Effect of Core Self-Evaluation and Attentional Control

**DOI:** 10.3390/bs14121183

**Published:** 2024-12-11

**Authors:** Hongyan Shen, Tiansheng Xia, Shimin Fu

**Affiliations:** 1 Department of Psychology and Center for Brain and Cognitive Sciences, School of Education, Guangzhou University, Guangzhou 510006, China; 2 Mental Health Education and Counseling Center, the Student Affairs Office, Guangzhou University, Guangzhou 510006, China; 3 School of Art and Design, Guangdong University of Technology, Guangzhou 510090, China

**Keywords:** intolerance of uncertainty, core self-evaluation, attentional control, social anxiety, university students

## Abstract

Social anxiety is a serious and prevalent psychological problem among university students, with intolerance of uncertainty playing an important role in its formation and development. The underlying mediating processes remain elusive despite the existing research on the association between these two constructs. This investigation developed a sequential mediation model grounded in the triadic reciprocal determinism theory to examine the intermediary roles of core self-evaluation and attentional control. Utilizing a convenience sampling method, a total of 1580 undergraduate students were recruited for this study. The study variables were assessed using scales measuring intolerance of uncertainty, core self-evaluation, attention control, and social interaction anxiety. The results revealed a significant and positive predictive relationship between intolerance of uncertainty and social anxiety (effect = 0.10; SE = 0.02; 95% CI = [0.00, 0.05]; *p* < 0.001). Intolerance of uncertainty directly and indirectly impacted social anxiety via three pathways: the independent mediating influence of core self-evaluation (effect = 0.15; SE = 0.02; 95% CI = [0.12, 0.18]; *p* < 0.001), the independent mediating influence of attentional control (effect = 0.03; SE = 0.01; 95% CI = [0.02, 0.05]; *p* < 0.001), and a serial mediation effect involving both core self-evaluation and attentional control (effect = 0.04; SE = 0.01; 95% CI = [0.03, 0.06]; *p* < 0.001). These direct and indirect effects contributed 30.03% and 69.97% to the overall effect, respectively. This study offers novel insights for interventions and treatments targeting social anxiety in university students.

## 1. Introduction

Social anxiety, one of the most prevalent forms of anxiety [1], refers to the emotional responses of discomfort, tension, embarrassment, and fear experienced by individuals in anticipated or actual social situations, stemming from concerns about negative evaluation by others, an excessive preoccupation with social consequences, and an inability to effectively manage these feelings [2]. According to Erikson’s psychosocial development theory, university students are at the intersection of adolescence and early adulthood, a phase characterized by identity crises and uncertainty in interpersonal relationships [3]. These psychological developmental features may render this cohort particularly susceptible to social anxiety. Previous research has indicated that individuals at this stage exhibit high interpersonal sensitivity, frequently experiencing elevated levels of social anxiety, and in some cases, even extreme fear of normal social interactions [4]. A cross-national study spanning seven countries revealed that the prevalence of social anxiety among young adults ranges from 23% to 58% [5], with 87.8% of Chinese university students experiencing varying degrees of social anxiety [6,7]. Social anxiety undermines the physical and mental health of university students [7], diminishes individual happiness, impairs academic and social performance [8], and elevates the risk of developing depression and suicidal ideation and behaviors [9,10]. Given the high incidence and impact of social anxiety in collegiate students, it is, consequently, important to delve into the factors affecting social anxiety and its internal mechanisms and promote the prevention and management of social anxiety to ensure students’ physical and mental health.

In line with Bandura’s reciprocal determinism theory, prior studies have established a causal link between intolerance of uncertainty (IU) and the emergence and progression of anxiety disorders, suggesting a cyclical process that perpetuates individual anxiety levels [11,12]. Intolerance of uncertainty, a personality trait where one is particularly sensitive to the environmental context, often leads to negative emotional responses such as increased anxiety and behavioral avoidance strategies when faced with uncertain situations [13]. This theory posits that individuals’ behaviors, personal factors, and environmental influences are inextricably linked. IU has been identified as a major source of stress in life [14] and has been found to continuously increase over time in both North American [15] and Chinese university student populations [16]. Those who struggle with IU may exhibit more introverted, pessimistic, and avoidant behaviors in social contexts, which are behavioral responses influenced by their cognitive evaluations of uncertainty [17,18,19]. Direct evidence indicates that IU can profoundly disrupt social interactions and heighten social anxiety [20,21]. IU is pivotal in the shaping and progression of social anxiety, manifesting as a notable precursor with a substantial and positive predictive relationship [22,23,24,25]. While the relationship between IU and social anxiety has been documented, the intricate interplay between various factors remains underexplored. Elucidating these mechanisms is pivotal for devising effective anxiety reduction strategies for this demographic. Cognitive factors have been identified as contributors to the development and persistence of social anxiety [26]. Individuals with social anxiety often harbor maladaptive thoughts and beliefs, interpreting uncertain social situations as threatening, which is a cognitive bias manifest in IU. This bias leads to the overestimation of negative outcomes and subsequent rumination that consumes mental resources and provokes social avoidance and, thereby, anxiety [27]. This avoidance, a behavioral coping mechanism, reinforces the anxiety cycle, illustrating the reciprocal relationship between personal behaviors and cognitive processes. According to reciprocal determinism, such patterns interact with the social environment, creating a feedback loop. Our findings, grounded in previous research, suggest that when individuals are confronted with uncertain social situations they cannot tolerate, the resultant anxiety may erode their core self-evaluations [27] and compromise attentional control [28,29], thereby intensifying social anxiety. Consequently, we propose that core self-evaluations and attentional control may mediate the association between IU and social anxiety. This hypothesis aligns with the reciprocal determinism theory’s emphasis on the transactional influence of cognitive, behavioral, and environmental factors in the etiology and maintenance of psychological phenomena, offering a comprehensive understanding of the dynamics underlying social anxiety.

### 1.1. The Mediating Effect of Core Self-Evaluation

Core self-evaluation, defined as the fundamental assessment and perception of one’s own worth and competence, is an important personal factor within the complex personality construct. It is the central element in core evaluations, encompassing facets such as self-esteem, general self-efficacy, locus of control, and emotional stability [30]. The cognitive model of social anxiety underscores negative self-perceptions and evaluations as crucial contributors to the onset and progression of social anxiety [31]. Previous research has shown that core self-evaluation negatively predicts social anxiety: the lower the core self-evaluation, the higher the level of social anxiety [32,33]. Individuals with low core self-evaluation tend to be more receptive to negative information about themselves in social situations, which, in turn, reinforces their negative cognition and evaluation of the self, leading to the manifestation of increased anxiety [34]. In line with Bandura’s reciprocal determinism theory [35], this dynamic suggests that core self-evaluation is not only influenced by personal cognitive processes but also by the reciprocal interactions with the social environment, which can amplify or mitigate the effects of intolerance of uncertainty on social anxiety. Notably, recent studies have also found that core self-evaluation can act as a buffer against stress and anxiety by influencing one’s coping strategies. High core self-evaluators are more likely to adopt problem-solving and adaptive coping strategies, which further mitigate the adverse effects of stress and anxiety [36,37].

Previous research has found a significant negative correlation between uncertainty tolerance and core self-evaluation [38,39]. Several studies have additionally demonstrated that a heightened intolerance of uncertainty can precipitate a decrement in the pivotal elements of core self-evaluation, such as reduced self-control or diminished evaluations of other forms of control [40] and decreased academic self-efficacy [41], causing higher levels of emotional instability [42,43] and lower self-esteem [44]. Furthermore, numerous studies have shown that these effects may arise regardless of the size of the probability of uncertain situations or events and their consequences [43,45,46,47]. Accordingly, this study formulated hypothesis H2: Core self-evaluation functions as a mediating factor in the relationship between intolerance of uncertainty and social anxiety.

### 1.2. The Mediating Effect of Attentional Control

Attentional control, a crucial behavioral strategy for managing and regulating emotional responses to uncertainty, involves the conscious activation, concentration, and maintenance of attention amidst distractions. It is characterized by the ability to concentrate intently, swiftly reorient attention, and adeptly modulate focus, thus enabling individuals to avoid excessive focus on threatening information. Importantly, attentional control has been found to be a significant factor in moderating the relationship between anxiety and environmental cues [37,48]. Individuals with a high IU are inclined to be hyperattentive and vigilant to environmental cues when facing uncertainty, which can hinder their ability to disengage from negative stimuli, exacerbating subsequent negative emotional responses [49,50]. This cognitive predisposition toward threat is associated with impaired attentional control, where an excessive focus on uncertain information contributes to heightened anxiety levels [51]. Moreover, such individuals often adopt maladaptive coping strategies, characterized by a reduction in attentional control, to manage their emotional experiences [52]. Conversely, effective attentional control is inversely related to social anxiety, with those exhibiting weaker control being less capable of detaching from threatening information, thus intensifying anxiety symptoms [29]. Attentional control is also a defensive mechanism, enabling individuals to avoid negative thoughts and emotional responses, thereby maintaining lower anxiety levels [28]. Research has shown that individuals with high core self-evaluations are more likely to cultivate effective attentional control strategies, enabling them to maintain focus and reduce distractions when facing tasks [53]. This is in line with the self-efficacy theory [54], which suggests that robust core self-evaluations lead to stronger self-confidence and a sense of internal control. Therefore, individuals with high core self-evaluations are better equipped to maintain attentional control, even in uncertain or stressful situations.

It could be hypothesized that core self-evaluation may elicit profound changes in emotional regulation strategies and psychological resilience when it is influenced by intolerance of uncertainty, which, in turn, potentially leads to impaired attention control functions and developing an attentional bias toward negative or threatening information, ultimately playing a pivotal role in initiating or amplifying instances of social anxiety [55]. Hence, this study proposed hypothesis H3: Intolerance of uncertainty impacts social anxiety via independent mediation by core self-evaluations and a sequential mediation involving both core self-evaluations and attentional control.

The triadic reciprocal determinism theory proposed by Bandura posits that an individual’s environment, personal factors, and behaviors interact and influence each other, forming a comprehensive interactive system that maintains a dynamic equilibrium. With core self-evaluations and attention control as variables of personal factors, this study explores the mechanism underlying the impact of uncertainty tolerance on social anxiety, taking the uncertain information perceived by individuals as an external environmental factor.

### 1.3. Research Purposes

**Figure 1:**
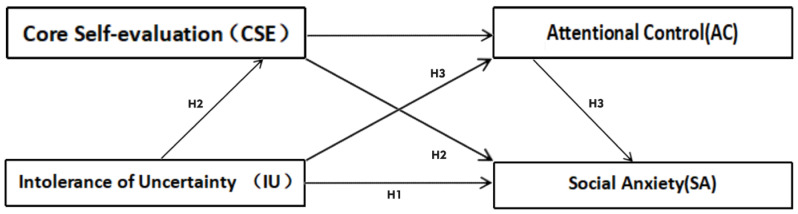
A predictive model of social anxiety among university students.

We constructed a sequential mediation model in this study (Figure 1). In line with Bandura’s reciprocal determinism theory, which posits that personal factors, behaviors, and environmental influences are in a dynamic interplay, this study aimed to investigate the multifaceted relationship between uncertainty intolerance and social anxiety, considering how individual factors and behaviors interact with environmental cues. Building upon the foundations laid by previous research, this study’s primary objectives encompassed three distinct facets: H1, uncertainty intolerance exerts a pronounced and positive influence on the manifestation of social anxiety; H2, core self-evaluation functions as a mediator, bridging the connection between uncertainty intolerance and social anxiety; and H3, the impact of IU on social anxiety is mediated by core self-evaluation and operates via a sequential mediating pathway involving both core self-evaluation and attentional control.

## 2. Method

### 2.1. Participants

This study’s participants were conveniently sampled, comprising 1580 students aged 16 to 25 years (M = 19.53; SD = 1.23) recruited from three public universities. These institutions were chosen to offer a cross-section of academic specializations, including natural sciences, humanities, and interdisciplinary studies, thereby ensuring a broad representation of the academic landscape. The participants completed their questionnaires in class under the supervision of researchers in May 2024. In total, 1580 valid questionnaires were collected, among which 584 were from male (37%) and 996 from female (63%) students. The Ethics Committee of the Psychology Department, Guangzhou University, approved this study. Prior informed consent was secured from all participating individuals.

### 2.2. Measures

#### 2.2.1. Interaction Anxiety Scale

This study utilized the Chinese version of the Interaction Anxiety Scale [37], previously validated for use among Chinese students, to gauge the prevalence of interaction anxiety among university students, with good reliability and validity [48,56]. This scale encompasses 15 items including “I often feel nervous even at casual gatherings”, rated on a 5-point Likert scale ranging from “strongly disagree” (1) to “strongly agree” (5). This study yielded an internal consistency coefficient of 0.84 for the scale.

#### 2.2.2. Intolerance of Uncertainty Scale

We utilized the Intolerance of Uncertainty Scale [36], validated for its reliability and appropriateness in research on Chinese students [57,58], to assess the extent of uncertainty intolerance among the student cohort. This scale comprises 12 items, scored on a 5-point Likert scale ranging from “completely disagree” (1) to “completely agree” (5). Our findings revealed an internal consistency coefficient of 0.84 for this scale.

#### 2.2.3. Core Self-Evaluation Scale

Subsequently, we administered the Core Self-Evaluation Scale [59], which has been translated and refined and has been extensively used in research on Chinese students, demonstrating robust reliability and validity [60,61]. Its 10 items are rated on a 5-point Likert scale, with responses ranging from “strongly disagree” (1) to “strongly agree” (5). Higher scores on the scale are indicative of a stronger core self-evaluation. In this study, the scale yielded an internal consistency coefficient of 0.86.

#### 2.2.4. Attention Control Scale

The Attention Control Scale measures an individual’s ability to shift or concentrate attention across different situations [55]. The Chinese adaptation of the scale was translated and revised by Yang [62], with good reliability and validity among Chinese university student populations [63]. A higher score on this scale indicates superior attentional control. It comprises 20 items, such as “In a noisy environment, I find it difficult to focus and complete a difficult task”. The Likert 4-point scoring method was applied, ranging from “strongly disagree” to “strongly agree”. The internal consistency coefficient for this scale was 0.78 in this study.

### 2.3. Analytical Method

The statistical analysis was conducted utilizing SPSS 25.0 and the PROCESS v3.4. Initially, common method bias was assessed using exploratory factor analysis, followed by determining the mean and standard deviation for each variable and performing Pearson correlation analyses. Lastly, the bias-corrected nonparametric percentile bootstrap method [64] was applied to assess the mediated effects. Hayes’ PROCESS macro model 6 was employed to precisely estimate serial mediation, which involved a resampling procedure with 5000 iterations to establish a 95% confidence interval for the mediating effects.

## 3. Results

### 3.1. Common Method Bias Test

An exploratory factor analysis was performed to assess the presence of potential common method bias [65]. The findings indicated that nine factors had eigen root values exceeding one. Furthermore, the first common factor explained merely 21.25% of the cumulative variance, which was below the commonly accepted threshold of 40.00%. These findings indicated that the data in this study were not substantially affected by common method bias.

### 3.2. Descriptive Statistics and Correlation Analysis

Pearson correlation analysis was employed to examine the potential linkages between interaction anxiety, intolerance of uncertainty, core self-evaluation, and attention control. Table 1 offers a comprehensive portrayal of the descriptive statistics for each variable. The analysis revealed that interaction anxiety was significantly positively correlated with intolerance of uncertainty and significantly negatively correlated with core self-evaluation and attention control (*r* = −0.30, −0.49, and −0.44; *p* < 0.001). Intolerance of uncertainty, in turn, displayed significant positive correlations with core self-evaluation and attention control (*r* = −0.42 and −0.30; *p* < 0.001). Moreover, core self-evaluation exhibited a significant positive correlation with attention control (*r* = 0.47; *p* < 0.001). Given the significant correlations observed between gender and the key variables addressed in this study, gender was incorporated as a control variable in the subsequent data analyses.

### 3.3. Mediating Effect Test

Gender was included as a covariate in the mediation effect analysis to enhance the precision of our findings. As is evident in Table 2, in the regression analysis, intolerance of uncertainty emerged as a significant positive predictor of social anxiety (*β* = 0.10; *p* < 0.001), supporting H1, while concurrently exhibiting a significant negative influence on core self-evaluations (*β* = −0.31; *p* < 0.001), which confirms H2. Core self-evaluation, in succession, demonstrated a robust positive predictive relationship with attentional control (*β* = 0.42; *p* < 0.001), underscoring its pivotal role and supporting H3. We observed the following salient effects when collectively examining all variables within the regression model: intolerance of uncertainty maintained its significant and direct predictive power over social anxiety (*β* = 0.10; *p* < 0.001), reaffirming H1; conversely, core self-evaluations (*β* = −0.48; *p* < 0.001) and attentional control (*β* = −0.35; *p* < 0.001) significantly and negatively predicted social anxiety, providing further evidence for H3.

**Figure 2:**
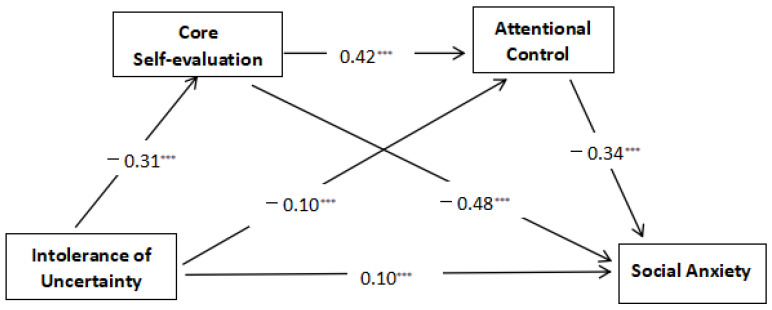
The final model illustrates the chain mediation of the associations between core self-evaluation and attentional control affecting intolerance of uncertainty and social anxiety. Note: *** *p* < 0.001.

The mediation model, as presented in Table 2 and Figure 2, comprised a single direct effect pathway alongside three mediating effect pathways. The breakdown of the total effect (0.33) revealed that the direct effect (0.10) contributed 30.03%, while the indirect effects (0.23) accounted for the remaining 69.97%. Within these indirect effects, path 2, which supports H2, held an effect value of 0.15, comprising 46.30% of the total effect (SE = 0.01; 95% CI = [0.12, 0.18]; *p* < 0.001); path 3 contributed 10.12% of the total effect, exhibiting an indirect effect value of 0.03 (SE = 0.01; 95% CI = [0.02, 0.05]; *p* < 0.001); and lastly, path 4 accounted for 13.55% of the total effect, with an indirect effect value of 0.04 (SE = 0.01; 95% CI = [0.03, 0.06]; *p* < 0.001), further supporting H3 by indicating the additional mediating pathway through attentional control.

## 4. Discussion

This study investigated the mediating roles of core self-evaluation and attentional control in the correlation between social anxiety and intolerance of uncertainty among university undergraduates. It delineated the mechanisms through which aversion to uncertainty influences the manifestation of social anxiety by modulating intrapersonal attributes within this cohort. An integrated theoretical analysis was conducted to elucidate the subtleties of the underlying dynamic processes and provide a conceptual framework for mitigating the deleterious repercussions of social anxiety in the collegiate population. The empirical results indicate that the association between aversion to uncertainty and social anxiety is complex, exhibiting both direct and indirect effects, with the latter being mediated via the distinct pathways of core self-evaluation and attentional control. These mediating effects manifest in three distinct pathways: (a) a unique mediating effect of core self-evaluation, (b) a separate mediating effect of attentional control, and (c) a conjoint mediating effect involving the intercession of both core self-evaluation and attentional control.

### 4.1. The Influence of Intolerance of Uncertainty on Social Anxiety in University Students

This study uncovered a significant positive association between intolerance of uncertainty and social anxiety in university students, corroborating findings from prior research. The results support the triadic reciprocal determinism theory and the cognitive–behavioral model [66]. That is, individuals who exhibit a strong aversion to uncertainty are inclined to excessively focus on potential negative outcomes or rejection when confronted with uncertain situations. This excessive focus, in turn, impedes their ability to promptly disengage from negative stimuli, thereby intensifying negative emotions. Consequently, these findings carry significant implications for undergraduate counseling services, suggesting that they should address intolerance of uncertainty as a key factor in managing social anxiety by incorporating strategies to help students develop resilience and coping skills.

A multifaceted academic discourse surrounds the interplay between intolerance of uncertainty and social anxiety. A contingent of researchers contends that social anxiety could potentially result from intolerance of uncertainty, whereas another school of thought posits that self-control is a pivotal mediator in this dynamic [67]. Furthermore, prior investigations have illuminated the concurrent presence of self-esteem, worry, and rumination, which collectively contribute to shaping the connection between these two constructs [44,67]. However, longitudinal studies that definitively establish causality between intolerance of uncertainty and social anxiety are absent. Thus, this unexplored terrain presents a promising avenue for future research endeavors.

### 4.2. The Mediating Role of Core Self-Evaluation

This research affirmed that core self-evaluation operates as a critical intermediary in the association between aversion to uncertainty and the manifestation of social anxiety, consistent with Bandura’s theory that personal factors such as self-evaluation can influence behavior and environmental outcomes. Specifically, intolerance of uncertainty decreases core self-evaluation by placing an excessive focus on negative self-related information and negative evaluations of oneself in uncertain situations, which, in turn, elevates the propensity for social anxiety. This conclusion aligns with prior research, which has consistently observed that intolerance of uncertainty leads to low core self-evaluation, and such reduced self-evaluation is a precursor to social anxiety [32,33]. This study simultaneously integrated all three variables under investigation, revealing the pivotal mediating function of core self-evaluation in the influence of intolerance of uncertainty on socially anxious behaviors. According to the cognitive model of social anxiety, behavioral responses are markedly influenced by self-perceptions. Individuals with a high degree of intolerance for uncertainty, particularly university students, exhibit lower levels of core self-evaluation. This diminished self-evaluation may heighten an individual’s dependency on maladaptive coping strategies to manage social-situation-related anxiety [68]. This suggests that undergraduate counseling should include interventions aimed at improving core self-evaluation among students with high intolerance of uncertainty, as this may help break the cycle of social anxiety and ineffective coping strategies. Consequently, these maladaptive strategies can amplify the detrimental effects of social anxiety, thereby perpetuating a vicious cycle of anxiety and ineffective coping mechanisms.

### 4.3. The Mediating Role of Attentional Control

This empirical inquiry indicates that attentional control operates as a mediating factor in the correlation between the propensity for intolerance of uncertainty and the experience of social anxiety among higher education students, aligning with the theory that cognitive processes can influence both behavior and environmental outcomes [54]. Specifically, an individual’s propensity for intolerance of uncertainty can precipitate social anxiety by diminishing their capacity for attentional control. In essence, attentional control emerges as an important mechanism through which intolerance of uncertainty impacts social anxiety within the collegiate population. The findings proffer an explanatory framework for understanding the psychological mechanism through which uncertainty intolerance contributes to social anxiety symptoms among collegians, focusing on the cognitive internalization of attentional processes. Consistent with the over-involvement theory [49], an excessive focus on threat-related information during ambiguous situations results in a deficit of cognitive disengagement and the adoption of maladaptive coping strategies, both of which are pivotal in perpetuating anxiety disorder. In other words, individuals with a high IU are prone to excessively dwelling on potential rejection or negative information, finding it difficult to disengage from such stimuli, which is in line with Bandura’s theory that cognitive processes can perpetuate behavioral patterns. This may lead to decreased or impaired attention, preventing the effective allocation of attentional resources for adaptive regulation. Consequently, this hinders the adoption of problem-solving strategies, further intensifying uncertainty and exacerbating social anxiety.

### 4.4. The Chain Mediation Impacts of Core Self-Evaluation and Attentional Control

This study further delineated that core self-evaluation and attentional control function as sequential mediators in the pathway from intolerance of uncertainty to social anxiety, reinforcing the established link between core self-evaluation and attentional control [69]. According to Bandura’s reciprocal determinism theory, these personal factors interact with environmental cues and behavioral outcomes, creating a cyclical relationship that influences anxiety levels. The obtained findings suggest that a higher propensity for intolerance of uncertainty is associated with an increased probability of experiencing social anxiety, a relationship modulated by a composite of self-esteem, control beliefs, general self-efficacy, and emotional stability—constituting core self-evaluation—alongside attentional control. As pivotal personal factors, core self-evaluation and attentional control underpin the formulation of cognitive and behavioral coping strategies utilized by individuals facing social anxiety. Furthermore, this study underscores the role of core self-evaluation and attentional control as both risk and maintenance factors in the etiology of individual anxiety levels. Individuals prone to a high intolerance of uncertainty are inclined to develop diminished core self-evaluations when encountering uncertain social scenarios that may result in rejection or adverse feedback, which adversely affects their attentional control. Such individuals may become preoccupied with negative self-relevant information and struggle to disengage their focus from these cues. Such individuals may become preoccupied with negative self-relevant information and struggle to disengage their focus from these cues, reflecting the interplay of personal and cognitive factors as described by Bandura. This impaired ability to self-regulate and shift attentional focus can ultimately precipitate the onset of social anxiety. Given these findings, undergraduate counseling services should prioritize interventions aimed at enhancing core self-evaluation and attentional control among students with high intolerance of uncertainty. By strengthening these psychological mechanisms, counselors can help students develop more effective coping strategies, thereby mitigating the symptoms of social anxiety.

In the syntactic analysis of the extant literature concerning the mediating role of social anxiety, the observed effect sizes spanned a broad spectrum from 46.81 to 100% [44,67,70], each offering innovative intervention vectors for prognosticating the socio-behavioral patterns of tertiary education students and attenuating the prevalence of social anxiety. The mediating effect ascertained in this empirical inquiry was relatively diminished, which corroborates the empirical outcomes of antecedent studies [70]. Nonetheless, the findings substantiate the conceptual validity of the theoretical framework, indicating that core self-evaluation and attentional control are proximal psychological mechanisms that partially delineate the correlation between the propensity for intolerance of uncertainty and the incidence of social anxiety. In effect, strategically amplifying core self-evaluation and attentional control to fortify self-efficacy and cognitive regulatory mechanisms in the context of adaptive coping strategies may be a salient factor in mitigating social anxiety symptomatology among the collegiate population.

### 4.5. Implications and Limitations

This study elucidates the role of intolerance of uncertainty in precipitating social anxiety among university students, elucidating the sequential mediating impacts of core self-evaluation and attentional control. The findings offer a conceptual foundation for the development of targeted interventions aimed at alleviating social anxiety in this demographic, while also highlighting the significance of mental health promotion during the university period. This research indicates that intolerance of uncertainty can significantly impair core self-evaluation and attentional control, leading to reduced social competence and the onset of social anxiety. Furthermore, previous research suggests that core self-evaluation is a critical determinant of psychological well-being in adolescence and youth [71]. Consequently, it is imperative for educational institutions and affiliated organizations to support students in nurturing positive core self-evaluations, enhancing attentional control, and embracing personal diversity to bolster their self-confidence and prevent the occurrence of social anxiety.

Nonetheless, the current study had some limitations that need to be improved in future research: Initially, this study utilized a cross-sectional design approach in examining intolerance of uncertainty and social anxiety, as well as the mechanisms that underlie them, which precludes the determination of causal relationships between these variables. Secondly, the data collection relied solely on self-report instruments, potentially subjecting the findings to biases stemming from participants’ reporting tendencies and memory recall. Thirdly, this study’s participants were all recruited from the same city, and the gender ratio was imbalanced. Future studies may consider broadening the sample to achieve a gender equilibrium. Lastly, there are many factors influencing social anxiety among undergraduate students, and future studies may consider incorporating more variables, such as self-esteem, social support, and cognitive biases, which are closely related to social anxiety, to conduct in-depth explorations of undergraduate students’ social anxiety and thereby provide insights into the formation mechanisms of social anxiety among university students, improve the mediation effect percentage of the mediation model, and offer new insights that will improve the physical and mental health of university students.

## 5. Conclusions

This research’s findings suggest that intolerance of uncertainty is a pivotal determinant in the manifestation of social anxiety among university students. The association between these constructs is moderated by core self-evaluation and attentional control. Promoting the development of positive core self-evaluation and enhancing attentional control in university students are conducive to alleviating social anxiety levels.

## Figures and Tables

**Table 1:** The mean, standard deviation, and correlation matrix of each variable (*n* = 1580).

Variables	M ± SD	1	2	3	4	5
1. Gender	-	-				
2. SA	51.51 ± 9.49	0.22 ***	-			
3. IU	32.12 ± 8.83	0.01	0.30 ***	-		
4. CSE	31.77 ± 6.56	−0.06 **	−0.49 ***	−0.42 ***	-	
5. ACS	49.80 ± 6.69	−0.09 ***	−0.44 ***	−0.30 ***	0.47 ***	-

Notes: ** *p* < 0.01 and *** *p* < 0.001.

**Table 2:** Mediating effects of core self-evaluation and attentional control on the relationship between uncertainty and social anxiety.

	Core Self-Evaluation			Attentional Control			Social Anxiety		
	b	SE	*t*	b	SE	*t*	b	SE	*t*
Constant	43.06	0.75	57.17 ***	40.98	1.30	31.51 ***	74.92	2.20	34.00 ***
Gender	−0.79	0.31	−2.55 *	−0.84	0.31	−2.76	3.37	0.41	8.29 ***
IU	−0.31	0.02	−18.39 ***	−0.10	0.02	−5.31 ***	0.10	0.02	3.95 ***
CSE				0.42	0.02	16.93 ***	−0.48	0.04	−13.46 ***
AC							−0.34	0.03	−10.05 ***
*R* ^2^	0.18			0.24			0.33		
*F*	172.81			163.30			195.31		

Notes: * *p* < 0.05, *** *p* < 0.001 SE: standard error.

## Data Availability

The data presented in this study are available upon request from the corresponding author.
